# Zadek Osteotomy, a Good Treatment Option for Refractory Haglund’s Deformity

**DOI:** 10.7759/cureus.39497

**Published:** 2023-05-25

**Authors:** Yiteng Xu, Zulfiqar A Haider, Vail Karuppiah, Sunil Dhar

**Affiliations:** 1 Trauma and Orthopaedics, Torbay Hospital, Torbay and South Devon NHS Foundation Trust, Torquay, GBR; 2 Trauma and Orthopaedics, Nottingham City Hospital, Nottingham University Hospitals NHS Trust, Nottingham, GBR

**Keywords:** manchester-oxford foot questionnaire, heel prominence, calcaneal pitch angle, fowler-philip angle, haglund's deformity, zadek osteotomy

## Abstract

Introduction: Haglund's deformity (a prominence in the posterosuperior aspect of the calcaneum) is a known cause of posterior heel pain. Surgery is reserved for patients after failed conservative treatment. Zadek osteotomy is a dorsal-closing wedge osteotomy that reduces the posterior heel prominence. Zadek osteotomy is becoming a favored procedure, however, there are still relatively few studies focusing on patient-reported outcomes. Our main aim was to assess patient-reported outcomes following the Zadek osteotomy in refractory Haglund’s deformity. Our secondary aim was to evaluate the correlation between patient outcomes and changes in their pre and postoperative Fowler-Philip and calcaneal pitch angles.

Methods: We conducted a retrospective review of 19 patients (20 heels) who underwent Zadek osteotomy by a single surgeon at a tertiary hospital over six years. Patient-reported outcomes were collected preoperatively and at 12 months postoperatively using the validated Manchester-Oxford foot questionnaire (MOXFQ) scoring system. We also calculated the difference in their pre and postoperative Fowler-Philip angles and calcaneal pitch using the picture archiving communication system.

Results: There was an average improvement of 108 points in the MOXFQ score at 12 months (P<0.05). There was no statistically significant change in calcaneal pitch. However, the Fowler-Phillip angle dropped with an average of 11.4 º (P<0.05). A decrease in the Fowler-Philip angle does improve patient-related outcome measurement scores, however, the relationship is not directly proportional with "r" measured at 0.23.

Conclusion: Our results show that Zadek osteotomy is a useful procedure to consider in patients with symptomatic refractory Haglund’s deformity, with an improvement in patient outcomes at 12 months. However, further studies are needed to give stronger evidence for the efficacy of this procedure and its radiological correlations.

## Introduction

Haglund's deformity was first described by Swedish orthopaedic surgeon Patrick Haglund in 1927 [1]. The deformity refers to the prominence in the posterosuperior aspect of the calcaneum. It often develops as a result of rubbing and irritation of footwear. This causes impingement of the retrocalcaneal bursa and/or tendo Achilles (TA) insertion, producing a painful and inflamed heel [2].

Treatment options include conservative management and surgical intervention. Surgery is reserved for patients with refractory symptoms. The most common symptoms were continued pain and swelling at the site. Zadek was an American orthopaedic surgeon who first described the procedure in 1939 [2]. It is a dorsal closing-wedge osteotomy, which tilts the heel prominence anteriorly. It is one of the two main surgical solutions with more advantageous patient outcomes (the other being resection of the prominence) [3,4].

Haglund’s deformity can be radiologically quantified using Fowler-Philip and calcaneal pitch angles. However, there is sparse literature on its correlation with radiographic formulations. Therefore, we set out to examine the effectiveness of this procedure with the following aims: 1) patient-reported outcome measures following Zadek osteotomy using the Manchester-Oxford foot questionnaire (MOXFQ) preoperatively and at 12 months postoperatively; 2) evaluate changes in calcaneal angles postoperatively and investigate the correlation with patient outcomes.

## Materials and methods

We conducted a retrospective review involving 19 patients (20 heels) who underwent Zadek osteotomy for Haglund’s deformity for six years. It was a single-centre, single-surgeon study. All patients had at least six months of conservative treatment which entailed modification of footwear and physiotherapy sessions. Those who failed the first-line treatment were then selected for inclusion in the study.

Haglund’s deformity was clinically and radiologically confirmed by examination and plain radiographs, respectively. All patients had an MRI for preoperative planning and to rule out other causes of posterior heel pain. The inclusion and exclusion criteria are outlined in Table 1.

**Table 1:** Inclusion and exclusion criteria of the study

Inclusion criteria	Exclusion criteria
Age >18	Age <18
Haglund’s deformity confirmed by clinical examination and plain radiographs	Zadek osteotomy performed for other causes
Failed conservative management	Patients who did not consent

All patients had a lateral view, and weight-bearing radiographs pre and postoperatively. We calculated the change in their Fowler-Philip and calcaneal pitch angles using standardised measuring tools on the picture archiving communication system (PACS). We then quantified the patients’ preoperative and 12th month postoperative symptoms using the MOXFQ questionnaire. The data were analysed using Student’s t-test and Pearson product-moment correlation coefficient r.

Surgical procedure

**Figure 1:**
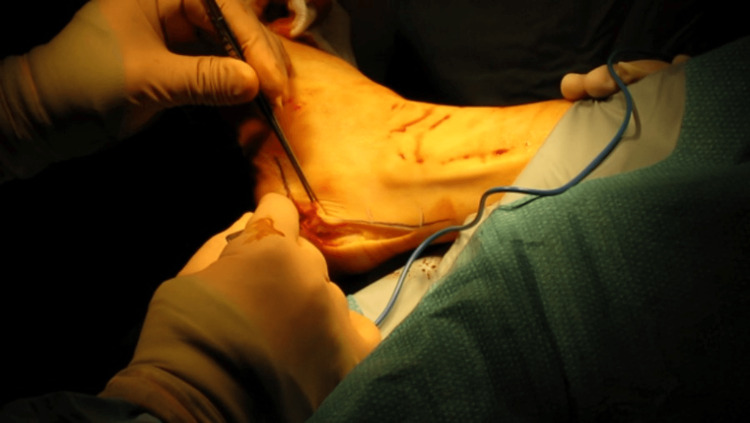
An extended lateral approach was used to expose the site of the osteotomies. The TA is not visualised or debrided. TA: Tendo Achilles

The procedure was originally described by Isadore Zadek in his paper on three women with achillobursitis, because of “failures encountered after the usual surgical procedures” for this condition [2]. We used a modified approach of the original method via an extended lateral approach. The incision was made along the anterior border of the TA and the flap was elevated (Figure 1), but the TA itself was not visualised or debrided. The posterior superior prominence of the calcaneal tuberosity was then exposed and sites of osteotomies were identified. At the apex, just posterior to the attachment of the plantar fascia to the calcaneum is the first landmark. Under image guidance, a vertical osteotomy perpendicular to the plantar surface was made, preserving the plantar ridge. This was followed by a second osteotomy between 7mm to 10mm (depending on the size of the "heel bump") from the previous one to remove a small wedge from the superior surface of the calcaneum. The osteotomy gap was closed by dorsiflexion of the ankle and fixed using a two-hole one-third tubular plate.

**Figure 2:**
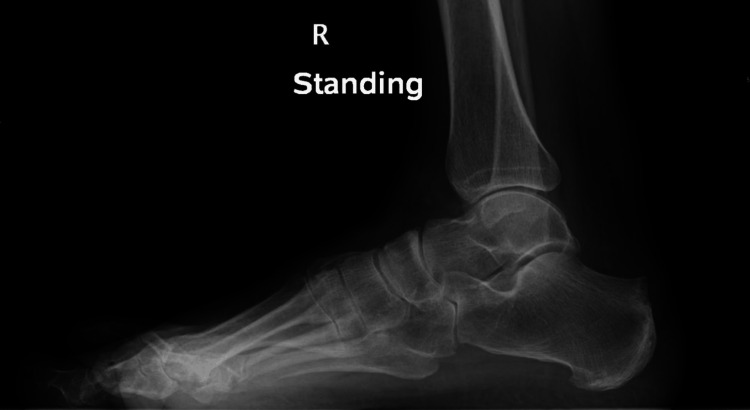
Preoperative radiograph of a symptomatic patient with Haglund’s deformity

**Figure 3:**
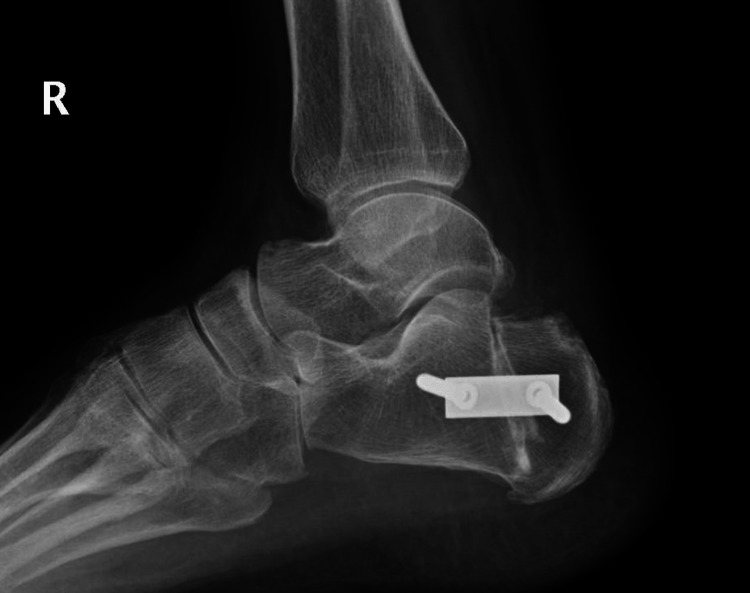
Postoperative radiograph of the same patient after Zadek osteotomy. The posterior prominence has been reduced, and the prominence has been tilted anteriorly.

The osteotomy aims to tilt the heel prominence anteriorly, which reduces the posterior prominence (Figures 2-3). It also slightly elevates the insertion of the TA and creates an effect similar to that of Achilles tendon lengthening [5]. It has been postulated that the orientation of the fibres at the calcaneal insertion is altered and hence reduces stress, which then reduces pain caused by insertional tendinopathy [6].

All patients had standardised postoperative management for weight bearing and footwear as follows: one to six weeks non-weight bearing in plaster; six to 12 weeks weight-bearing in a surgical boot; >12 weeks of normal footwear and return to usual activities. Follow-up appointments were conducted two weeks postoperatively for a wound check, and then at six weeks for plaster removal. The patients were subsequently discharged, and all had their MOXFQ administered telephonically 12 months postoperatively.

## Results

A total of 19 patients (20 heels) underwent Zadek osteotomy. All patients were fit and well, and independently mobile. The average age was 53 (range: 24-74) years, with 12 females and seven males. One patient was not contactable after the procedure, and another patient’s preoperative radiographs were not available to view on PACS. Therefore, their measurements were not included in our calculations, thereby generating a total of 18 heels in 17 patients (12 right and six left). There were no surgical complications in the immediate/early postoperative period. The data were analysed using the Student’s t-test in Microsoft Excel (Microsoft Corp., Redmond, WA, USA). The level of statistical significance was set at 5%.

Manchester-Oxford foot questionnaire

The MOXFQ is a patient-reported summary index scoring system widely used for evaluating outcomes in foot and ankle surgery (see Appendices). It was developed by Dawson et al. from an older questionnaire (Manchester Foot Pain and Disability Questionnaire) in 2006 [7]. It comprises three dimensions (pain, walking/standing, and social interaction), each with a maximum score of 64. The scores are summed from each dimension, and then converted to a metric score out of 100, generating a total score of 300. A higher score indicates worse symptoms.

Table 2 shows the average pre and postoperative metric scores within each dimension. The postoperative scores were collected 12 months following their procedure. There is good symptomatic improvement in each dimension (P<0.05).

**Table 2:** Displayed are the average pre and postoperative metric scores within each dimension of MOXFQ. The postoperative scores were collected 12 months following the procedure. MOXFQ: Manchester-Oxford foot questionnaire

Parameters	Preoperative	Postoperative	Average change
Foot pain (max 100)	66	27	↓39
Walking/standing (max 100)	59	24	↓35
Social interaction (max 100)	57	23	↓34
Total (max 300)	182	74	↓108

Angles

**Figure 4:**
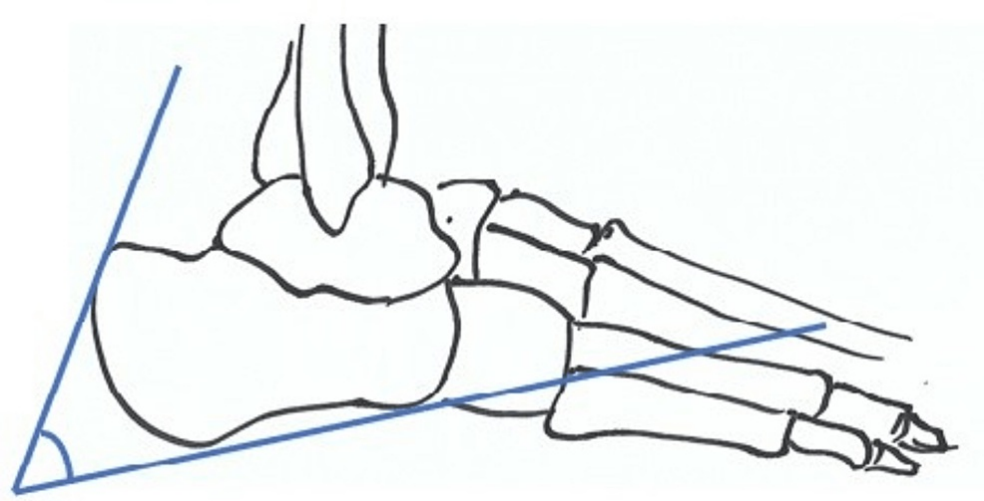
The Fowler-Philip Angle The schematic drawing of the foot illustrating the Fowler-Philip angle is the original work of the first author.

The Fowler-Philip angle was described by Fowler and Philip who studied 45 dry bones in 1945 [8]. This angle is derived from a line drawn between the posterosuperior prominence of the bursal area and the anterior tubercle and medial tuberosity (Figure 4). The normal range is between 44 to 69 degrees [8]. Angles >75º are associated with soft tissue pathology of the posterior heel [8].

**Figure 5:**
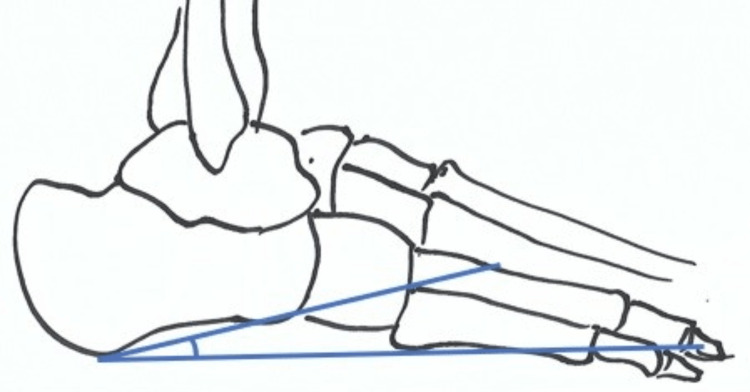
Calcaneal pitch The schematic drawing of the foot illustrating the calcaneal pitch is an original work of the first author.

The calcaneal pitch is calculated by first drawing a line along the anterior inferior surface of the calcaneum, extending from the calcaneocuboid joint to the plantar heel prominence. The second line is drawn from the plantar heel prominence to the fifth metatarsal base on a lateral weight-bearing radiograph (Figure 5) [9].

The average postoperative Fowler-Philip angle dropped by 11.4 degrees (range: 2-27) degrees, P<0.05 (see Table 3). The average calcaneal pitch decreased by 1.3 (range: -15-13) degrees, P=0.43.

**Table 3:** Average pre and postoperative Fowler-Philip and calcaneal pitch angles, respectively, showing a drop as indicated by downward arrows.

Calcaneal angles	Preoperative	Postoperative	Average change
Fowler-Philip angle	58.6º	47.2º	↓ 11.4º
Calcaneal pitch	27.8º	26.5º	↓ 1.3º

## Discussion

In our retrospective review, we found that Zadek osteotomy is an effective and safe procedure in our cohort of patients with refractory Haglund’s deformity. The MOXFQ questionnaires showed good improvement in symptoms one year after the osteotomy. We also found that a decrease in postoperative Fowler-Philip angle is consistent with an improvement in MOXFQ score.

Analysis of MOXFQ results

As a whole, patients had good improvement in systems across all domains (as shown in Table 2). The biggest improvement is in the pain domain (average decrease of 39), followed by walking/standing (35), and social interaction (34). The pain scores of three patients were less than the average change (between 10 to 25). It is noteworthy that of these patients who failed to improve, two had pre-existing plantar fasciitis. All patients had returned to their normal activities by the 12th-month follow-up questionnaire.

Fowler-Philip angle and patient outcome

There is limited literature on the relationship between radiographic formulations and the diagnosis of Haglund’s deformity. Several case-control studies, including those by Bulstra et al. and Lu et al., found no significant difference in the Fowler-Philip angle between symptomatic patients and controls [10,11]. Furthermore, Heneghan et al., Fuglsang (&) Torup, and Ruch all observed a large percentage of false negatives between the angle and symptomatic heels in their cohort of patients (100%, 86%, and 86%, respectively) [12-14]. Similarly, Kang et al. did not find a statistically significant correlation between radiological measurements and patient symptoms between the study and control group [15]. We only had one patient with a Fowler-Philip angle larger than 75 degrees preoperatively, although all were symptomatic. This is, again, in keeping with the results of the above studies.

**Figure 6:**
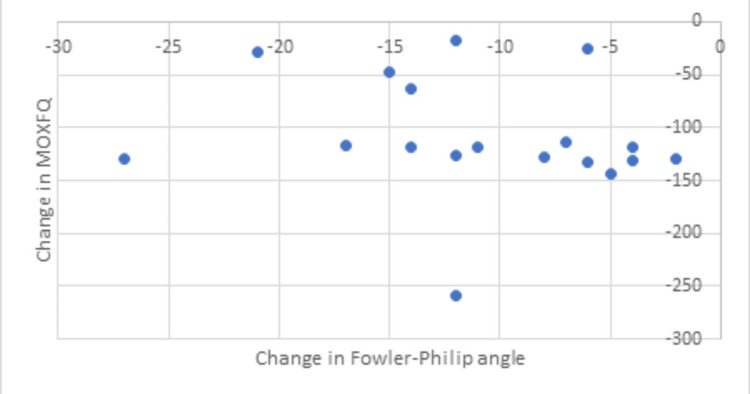
Scatter plot showing a decrease in the Fowler-Philip angle and improvement of MOXFQ score postoperatively MOXFQ: Manchester-Oxford scoring system

Zadek osteotomy reduced the Fowler-Philip angle by an average of 11.4 degrees (Table 3) in our patients. A decrease in the Fowler-Philip angle is consistent with an improvement in the MOXFQ score. However, it does not seem to be a strong linear relationship, i.e., a greater change in the Fowler-Philip angle does not generate better symptom improvement. The Pearson product-moment correlation coefficient r is 0.23. Figure 6 shows the change in MOXFQ score versus the change in the Fowler-Philip angle as a scatter plot rather than a linear relationship. It also illustrates that the majority of patients had good symptom improvement (a drop of between 100 to 150 in MOXFQ) following a decrease in the Fowler-Philip angle of up to about 20 degrees.

Calcaneal pitch angle and patient outcome

The normal calcaneal pitch angle is between 17 to 32 degrees [9]. It is thought that a large calcaneal pitch angle implicates verticalisation of the calcaneus, which causes further traction on the TA. Therefore, Bulstra et al. suggest that it is the vertical inclination that is more important than the absolute Fowler-Philip angle [10]. Singh et al. found a sensitivity of 29.6%, a false positive of 30%, and a false negative of 70.4% using calcaneal pitch in their symptomatic patients [9]. Thus, they concluded that a high arch foot alone does not lead to posterior heel pain. These findings appear to be consistent with our results: five of our patients had preoperative calcaneal pitch angles greater than 32 degrees, but the remaining were symptomatic despite a normal angle.

The calcaneal pitch decreased by an average of 1.3 degrees postoperatively (as shown in Table 3) in our patients. However, the change was not statistically significant, possibly due to a relatively small sample size. Therefore, it is not possible to draw conclusions about the relationship between the change in calcaneal pitch and MOXFQ scores. In a recent study, Tourne et al. found a statistically significant reduction in calcaneal pitch postoperatively [16]. Interestingly, they did not look into the clinical outcomes related to the Fowler-Philip angle, but it is important to note that they did report significant improvement in patient symptoms postoperatively after the Zadek osteotomy.

There does not seem to be a single angle that can accurately predict the severity of symptoms pre or postoperatively. This supports the theory that Haglund’s deformity can be caused by various clinical pathologies, rather than a single etiology-surgeons should be guided by the patient’s symptoms for the assessment and management of their clinical condition.

Future considerations

We acknowledge the limitations of the study: it is a retrospective study with a relatively small cohort. However, it is one of the largest studies to date with an intermediate-term follow-up and may act as a benchmark for other studies to judge their data. Further multi-centre prospective trials would be indicated with a longer follow-up to assess effectiveness in the long term. This would help develop more standardised guidelines for patients with Haglund’s deformity. It would also be interesting to further explore the role of radiographic formulations in patient outcomes.

## Conclusions

Zadek osteotomy is a safe and effective procedure for patients with refractory symptomatic Haglund’s deformity. It is reproducible and effectively decompresses the posterosuperior aspect of the calcaneus without the need for dissection around the Achilles insertion. It allows for consistent correction of Haglund's deformity, reliable symptom relief, and low rates of intra and postoperative complications. Furthermore, all patients in this study had improved outcomes at the 12th-month follow-up and returned to normal activity.

A decrease in the Fowler-Philip angle does improve patient-related outcome measurement scores, however, the relationship is not directly proportional. We recommend treating the patient's symptoms, whilst using the radiographic measurements as an aid. Further prospective multi-centre studies are needed to develop a consensus on universal radiological measurement tools and their clinical significance.

## Figures and Tables

**Figure 7:**
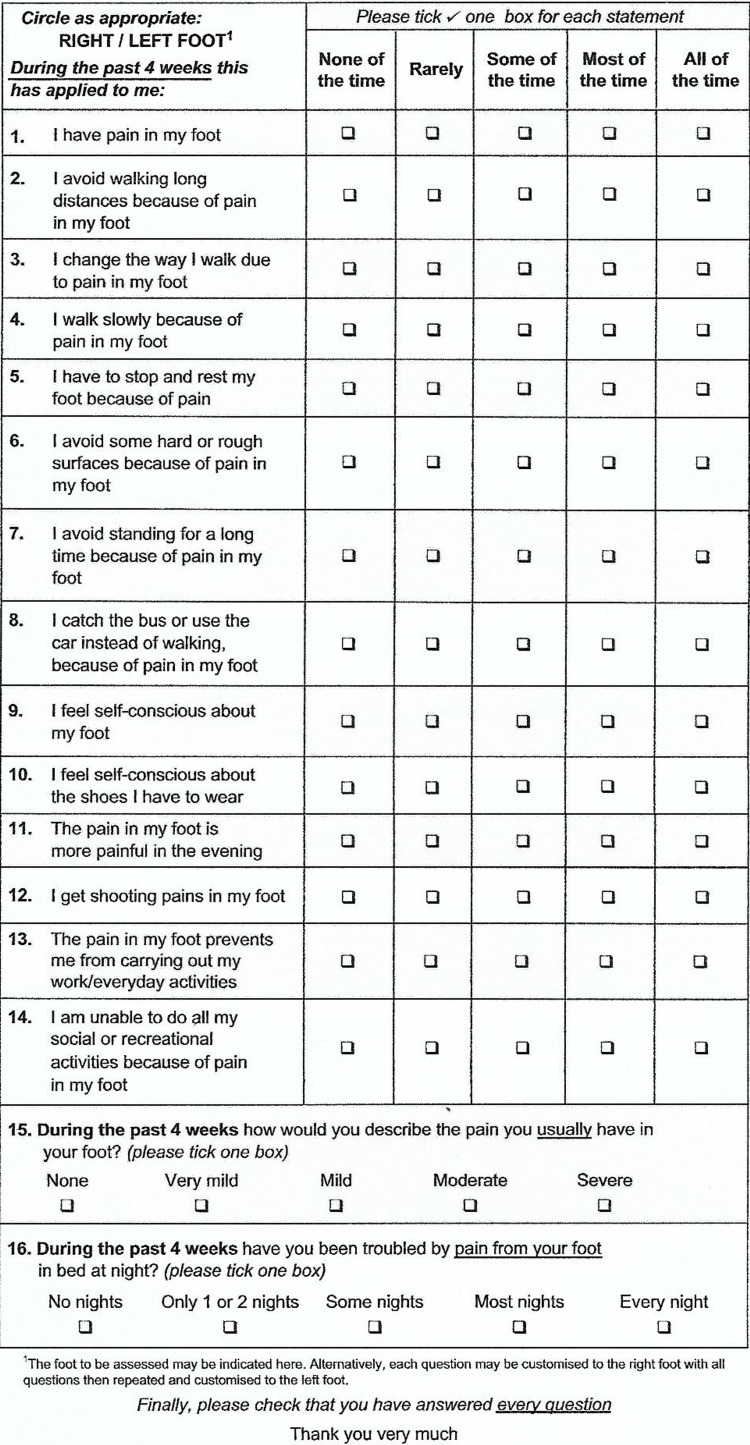
The MOXFQ is a patient-reported summary index scoring system widely used for evaluating outcomes in foot and ankle surgery MOXFQ: Manchester-Oxford foot questionnaire
